# Determinants of unmet need for contraceptive method among young married women in Ethiopia: Multilevel analysis of Ethiopia Demographic and Health Survey 2016

**DOI:** 10.1371/journal.pone.0306068

**Published:** 2024-09-05

**Authors:** Ebisa Turi, Galana Mamo Ayana, Sidise Temesgen, Adisu Tafari Shama, Bedasa Taye Merga, Tadesse Tolossa

**Affiliations:** 1 Department of Public Health, Institute of Health Sciences, Wollega University, Nekemte, Ethiopia; 2 School of Public Health, College of Health and Medical Science, Haramaya University, Harar, Ethiopia; 3 Department of Nursing, Institute of Health Sciences, Wollega University, Nekemte, Ethiopia; 4 Department of Public Health and Health Policy, School of Public Health, Haramaya University, Harar, Ethiopia; Ordu University, TURKEY

## Abstract

**Background:**

The notion of unmet need for family planning indicates the gap between women’s contraceptive practice and their reproductive intention. Although universal access to sexual and reproductive health services including contraceptive methods is a bedrock for sustainable development goals, the unmet need for contraception is high among young women in low-income countries including Ethiopia. The unmet need for contraception is associated with unintended pregnancy which most of the time end in unsafe abortion. Hence, this study aimed to assess the determinants of unmet need for family planning among young married women in Ethiopia using nationally representative data.

**Method:**

This study utilized secondary data collected in the 2016 Ethiopia Demographic and Health Survey (EDHS). A two-stage cluster sampling method was used. The analysis included a total of 2444 sexually active married young women (15–24 years). Multilevel logistic regression analysis was conducted to identify individual and community level factors associated with unmet need for contraceptives and the results were presented as adjusted odds ratio (AOR) at 95% confidence interval (CI), declaring statistical significance at a p-value <0.05 in all analyses.

**Results:**

In this study, the prevalence of unmet need for contraceptive method among married young women was 18.4% [95% CI: (16.9, 20.0)]. Female head of the household [AOR: 1.62, CI (1.25, 2.11)], primary level of education [AOR: 1.53, CI: (1.16, 2.03)], family size ≥5 [AOR: 1.53, 95%CI: (1.22, 1.93)], undecided to have child [AOR: 2.86, 95%CI: (1.58, 5.20)] and infecund [AOR: 1.54, 95%CI: (1.08, 2.20)] were factors positively associated with unmet need for family planning. Whereas the odds of unmet need for contraceptive method was lower among women-initiated sex between 15–17 years and >17 years [AOR:0.72, 95%CI (0.53, 0.98)] and [AOR: 0.58, 95%CI: (0.40, 0.85)] respectively and community with high proportion of poverty [AOR: 0.68, 95%CI: (0.46, 0.99)].

**Conclusion:**

The prevalence of unmet need for contraceptive methods among young married women was relatively high. Being female household head, age at first sexual intercourse, educational status of the woman, family size, desire for more children, and community poverty were significantly associated with unmet need for family planning. Hence, interventions targeting these special populations at the individual and community level would play a paramount role in meeting the unmet need for contraception among young married women in Ethiopia.

## Background

Unmet need for family planning is defined as the proportion of fecund, sexually active currently married or in-union women who desire to either postpone or limit childbearing, but who are not using any method of contraception [1]. The concept points to the discrepant behavior which is the gap between reproductive intentions and birth control practices [2]. Women are considered to have an unmet need for spacing if they do not want to become pregnant within the next two years while they are at risk of not using contraception. In another way, it is considered as an unmet need for limiting or terminating in case the women are at risk of becoming pregnant, not using contraception, and want no (more) children, pregnant with an unwanted pregnancy and postpartum amenorrhea for up to two years following an unwanted birth and not using contraception [1, 3, 4].

Young women are those women whose ages are found between 15 to 24 years [5]. Followed by migrant, urban slum dwellers, refugees, and women in the postpartum period, young women are one of the population groups in which unmet family planning need is high [2]. Evidence from different LMICs indicates that the unmet need of family planning for spacing is high among young married women aged 15–24 years as compared to women aged 25 and above [6, 7]. In Ethiopia, the contraceptive use among young currently married (15–24 years) women is 45.87% while the level of unmet need for young married women aged 15–19 years was higher (32.5%) than that for women between 40–44 and 45–49 years (24.1% and 14.4% respectively) [8].

Although universal access to Sexual and Reproductive Health (SRH) services including family planning methods is set as 2030 agenda in the Sustainable Development Goal (goal 3.7) [9], the unmet need for contraceptives is high in various countries [10–13]. In other developing countries, 11–38.2% prevalence of unmet need for contraception among young married women reported [11, 14–17]. Similarly, the prevalence of unmet need for family planning among young married women and women of reproductive age in Ethiopia is 21.6% and 34.6% respectively [18, 19]. Unmet need for spacing is more prevalent than the unmet need for limiting [20].

Family planning is an effective way to reduce fertility levels and lower future population growth which has positive impact on extending the human life span. Besides meeting the unmet need for contraceptive methods drops youth dependency and portends a demographic dividend, an added bonus to the already well-known benefits of meeting existing demands for family planning [21]. However, in Sub-Sahara African countries including Ethiopia, the level of unintended pregnancy among young women remained high which is an indication for unmet need for contraceptive methods [22]. Furthermore, childbearing at a very young age is associated with an increased risk of complications during pregnancy and childbirth and higher rates of neonatal mortality. Short birth intervals of less than 2 years can lead to health risks such as preterm birth, low birth weight, and death for both mothers and their newborns [8].

So far conducted studies identified that various factors like age, educational level, occupation, religion, residence, household wealth, knowledge about family planning methods and service availability, visit by a health worker, family planning services, and women empowerment are significantly associated with unmet need for contraceptive methods [10–13]. Misconceptions and concerns of the health risks associated with modern contraceptive methods keep the unmet need for family planning high among young married women [11, 20]. Unmet need of family planning is increased with the increased number of parities. Further, the likelihood of the unmet need for contraception is reduced by 30% for each additional child the married young women desire [18, 23].

Despite there are studies conducted on the unmet need for contraceptive method among reproductive women in Ethiopia, there is a paucity of studies to explore determinants of unmet need for family planning among young women (aged 15–24) at national level [24, 25]. Beside to this, young women are among the special population for unmet need of contraceptive method as identified by World Health Organization. However, most of studies conducted in Ethiopia are on the general population even though unmet need for family planning is high among young women. Moreover, the study conducted is limited to a specific locality which makes it insufficient to represent the country. Therefore, this study was designed to answer the research question: What are the determinants of unmet contraceptive needs among married young women in Ethiopia through multilevel analysis?

## Methods

### Data source

**Fig 1:**
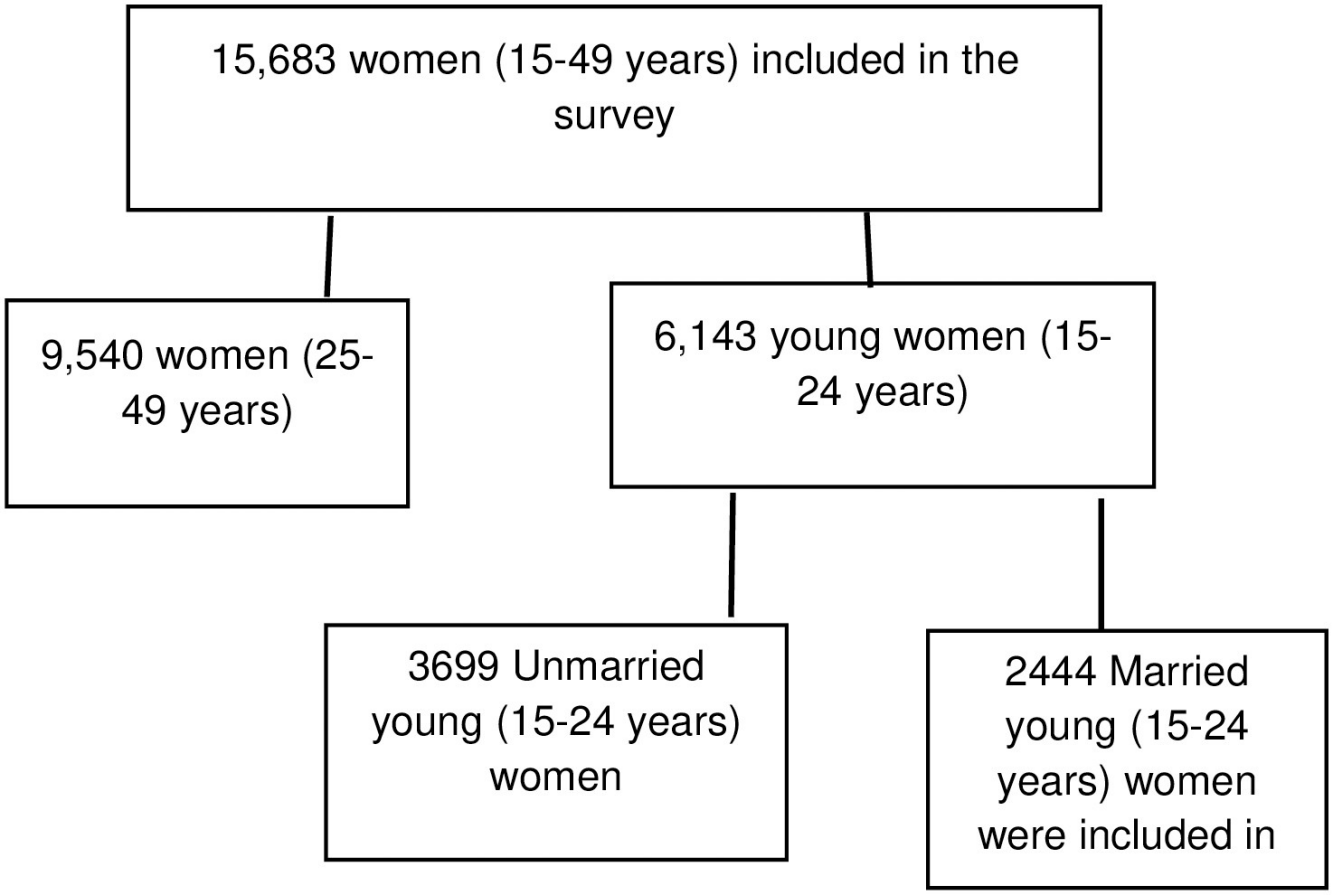
Sampling procedure and data extraction flow diagram for young married women in Ethiopia, EDHS 2016.

This study utilized secondary data collected in the 2016 Ethiopia Demographic and Health Survey (EDHS). The EDHS is nationally representative survey collected from nine regions and two administrative cities, from 18 January 2016 to 27 June 2016. A two-stage cluster sampling method was used. In the first stage, 645 clusters (202 urban areas and 443 rural areas) were randomly selected from the sampling frame (i.e., the 2007 Ethiopian population and housing census) and household listing. The second stage involved a systematic selection of 18008 households from the selected clusters, of which 17067 were occupied. Of the occupied cluster, 16650 were successfully interviewed. The information we used was related to young women of reproductive ages (15–24 years). A total of 15683 eligible women were identified for the survey. A total of 2444 sexually active young women (15–24 years) were included for analysis.

(Fig 1). Details about the DHS sampling techniques and datasets are available at (http://www.dhsprogram.com/).

### Inclusion and exclusion criteria

All married young women (15–24 years) in the selected household and completed individual interviews were included in the study.

### Variables of study

#### Outcome variable

The main outcome variable was the unmet need for contraception, where it composed of both unmet need for spacing and limiting form of unmet need. It refers to the proportion of women who desire to either delay the next pregnancy or limit future pregnancies but are not using any method of contraception. It was a binary variable, women with unmet need for spacing or limiting were recoded as ‘unmet need’, while those using contraception methods for spacing or limiting or with no unmet need were recorded as ‘no unmet need’ [26].

#### Independent variables

Independent variables included in the analysis are categorized as individual and community-level factors that are associated with unmet need for contraceptives.

#### Individual-level variables

Considered in the analysis were age, women’s level of education (no education, primary, secondary and higher), religion (recoded as Muslim, Orthodox, Protestant and others), education level of husband (categorized as no education, primary and secondary/higher), working status (not working/working), exposure to the media (categorized as ‘no’ if there is no media exposure at all and ‘yes’ if there is media exposure to either radio, magazine/newspaper, internet or television), wealth index (poorest, poorer, middle, richer and richest), number of living children, fertility desire (number of desired children categorized as “Has desire or has no desire”).

#### Community level variables

Community level variables were generated by aggregating the individual characteristics in a cluster since EDHS did not collect data that can directly describe the characteristics of the clusters except place of residence and region. The aggregates were computed using the proportion of a given variables subcategory within the given cluster. Since the aggregate value for all generated variables was not normally distributed. It was categorized into groups based on the national median values.

#### Community level media exposure

Aggregate frequency of watching TV, frequency of listening to radio, frequency of reading newspaper, of categorized as: <50% as low media exposed communities and >50% as high media exposed communities [27].

#### Community level of poverty

Aggregate wealth index of categorized as: <50% as high poverty communities and >50% as high poverty communities [27].

#### Place of residence

The variable place of residence recorded as rural and urban.

#### Region

It recorded nine regions and two administrative cities.

### Statistical analyses

Extracted data were weighted so that the sample was representative of 15–24-year-old respondents in 2016 EDHS. The importance of weighting data was to mitigate the effects of any sample imbalances. In addition, during the statistical weighting datasets are re-arranged through calculations in order to bring more in line with the population being studied. Analyses were performed using Stata version 14. Since, EDHS data has a hierarchical nature, women within one cluster may share similar characteristics together than women with different cluster. Due to this, the assumption of independent observations and equal variance across clusters might be violated. Therefore, considering advanced statistical models which can handle such data was unreplaceable to come up with a reliable standard error and unbiased model parameters. Multilevel logistic regression analysis was used to identify individual and community level factors associated with unmet need for contraceptives. Four models were considered in the multilevel analysis to determine the model that best fits the data; Model one: empty without explanatory variable developed to evaluate the null hypothesis that there is no cluster level difference in unmet need of contraceptives that specified only the random intercept and it presented the total variance in unmet need of contraceptives among clusters. Model two: adjusted for individual level variables which assume cluster level difference of unmet need for contraceptives is zero. Model three: was used to evaluate community level factor by aggregate cluster difference of unmet need for contraceptives. Multicollinearity assumption was checked using Variance inflation factor (VIF) of independent variables and variable with VIF less than 10 was considered as free of Multicollinearity. Model four: was used to include both adjusted individual and cluster level factors. The individual and community-level variables that determine unmet need for contraception were checked independently in the bi-variable multilevel logistic regression model and variables that were statistically significant at p-value 0.20 in the bi-variable multilevel logistic regression analysis were considered for the final individual and community level model adjustments. Finally, p-value less than 0.05 in the multivariable model of mixed-effects logistic regression was used to select variables which had statistically significant association with unmet need for family planning. The association was measured by adjusted odds ratio of individual and cluster level variable to identify factors that associate with unmet need of contraceptives. Measurement of variation which account for community level and individual level factors be identify using intraclass correlation (>10%,) [28] and proportional change in variance (PCV). The model for fitness diagnostics was selected by using Bayesian Information Criteria (BIC) or Akaike information criteria (AIC).

### Ethical approval and consent to participate

All DHS including EDHS used verbal informed consent for voluntary participation before interviewing participants. The 2016 EDHS data are available to the public by request in different formats from the Measure DHS website [http://idhsdata.org]. We applied the measure DHS by briefly stating the objectives of the study and got the permission to download the dataset in SPSS format. Permission letter for access to database was received from Measure DHS, ICF International, Rockville, Maryland, USA.

## Results

### Socio-demographic characteristics of young married women in Ethiopia

A total of 2444 married young women were included in the analysis. Most of the respondents 1,889 (77.3%) were from a rural setting. More than two-thirds of respondents 1782 (72.9%) were in the age group of 20–24 years. More than two-fifths of respondents 825 (35.9%) were not educated. Most of the respondents (66.4%) had cohabitation before age of 18 years (Table 1).

**Table 1:** Socio-demographic characteristics of young married women in Ethiopia, EDHS 2016 (n = 2444).

Variables	Categories	Frequency	Percentage
**Place of residence**	Urban	555	22.7
	Rural	1,889	77.3
**Age of respondents**	15–19	662	27.1
	20–24	1,782	72.9
**Age at first sex**	<15	438	17.9
	15–17	1,214	49.7
	> = 18	792	32.4
**Sex of household head**	Male	1,908	78.1
	Female	536	21.9
**Wealth index**	Poorest	832	34.0
	Poorer	381	15.6
	Middle	319	13.1
	Richer	286	11.7
	Richest	626	25.6
**Partner’s education level**	No education	825	33.8
	Primary	872	35.7
	Secondary	438	17.9
	Higher	284	11.6
	don’t know	25	1.0
**Respondents’ educational status**	No education	878	35.9
	Primary	1,090	44.6
	Secondary or above	476	19.5
**Family size**	<5	1,650	67.5
	> = 5	794	32.5
**Age at first birth**	Not given birth	710	29.1
	<18 years	796	32.6
	> = 18 years	938	38.4
**Age at first cohabitation**	<18 years	1,623	66.4
	> = 18 years	821	33.6
**Religion**	Orthodox	781	32.0
	Protestant	428	17.5
	Muslim	1,193	48.8
	Other	42	1.7
**Partners employment status**	Not working	212	8.7
	Working	2,232	91.3%
**Respondent employment status**	Not working	1,838	75.2
	Working	606	24.8

### Community level factors affecting unmet need for contraceptives

Nearly half of young women were from a high proportion of poor communities (49.5%) and high media exposed communities (50.2%). The highest proportion of young women were from Afar (13.2%) and the lowest was from Addis Ababa (4.1%) (Table 2).

**Table 2:** Community level factors for unmet need for contraception among young married women in Ethiopia, EDHS 2016 (n = 2444).

Variables	Categories	Frequency	Percentage (%)
**Place of residence**	Urban	555	22.7
	Rural	1,889	77.3
**Region**	Tigray	264	10.8
	Afar	322	13.2
	Amhara	225	9.2
	Oromia	316	12.9
	Somali	266	10.9
	Benishangul	202	8.3
	SNNP	237	9.7
	Gambela	209	8.6
	Harari	168	6.9
	Addis Adaba	100	4.1
	Dire Dawa	135	5.5
**Community Poverty level**	Low	1,235	50.5
	High	1,209	49.5
**Community Exposure**	Low	1,218	49.8
	High	1,226	50.2

### Unmet need, fertility desire

**Fig 2:**
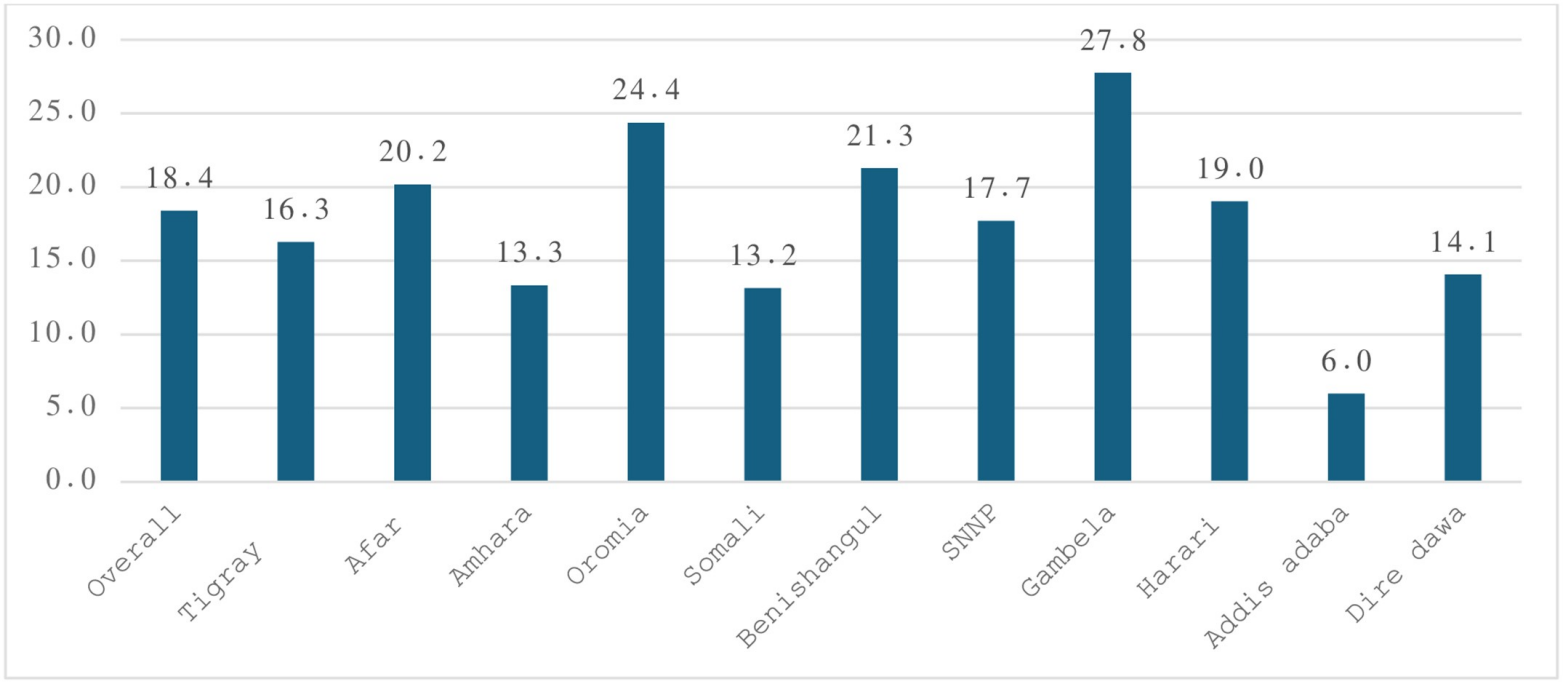
Unmet need among young married women by administrative regions and city councils in Ethiopia, EDHS 2016.

The overall unmet need for contraceptive among married young women (15–24 years old) was 18.4% (95%CI: 16.9,20.0). The unmet need for spacing and limiting was 89.3% and 10.7% respectively. Most of the respondents had knowledge about contraceptives (84.1%) (Table 3). Young women from Gambela regional state have the highest (27.8%) unmet need followed by Oromia (24.4%) and Benishangul (21.3%) regional states. Young women from Addis Ababa (6.0%) and Somali regional state (13.2%) councils have low unmet need for contraception (Fig 2).

**Table 3:** Unmet need for family planning, fertility desire, and contraceptive knowledge among married young women of Ethiopia, EDHS 2016 (n = 2444).

Variables	Categories	Frequency(n)	Percentage (%)
**Unmet need**	No	1,994	81.6
	Yes	450	18.4
**Type of Unmet need**	unmet need for spacing	402	89.3
	unmet need for limiting	48	10.7
**Knowledge about contraceptive**	Knows no method	388	15.9
	Knows modern contraceptive method	2,056	84.1
**Husband’s desire for children**	Has desire	1,595	65.2
	Has no desire	114	4.7
	don’t know	735	30.1
**Respondents Desire for more children**	Has desire	2,162	88.4
	Undecided	58	2.4
	wants no more or declared infecund	224	9.2

### Determinants of unmet need for family planning among young married women in Ethiopia

Prior to fitting the model Multicollinearity assumption was checked using Variance inflation factor of independent variables. Three different models (Null model, a model with individual level variables, and a model that contains individual level and community level variables) were fitted. The model that contains individual and community level variables was selected as the final model with relatively low AIC. The results of the empty model revealed that there was statistically significant variability in the odds of unmet need with community variance (τ = 0.60, p-value = p < 0.001). Similarly, the ICC in the empty model implied that a total of 13.5% variability in unmet need was attributed to the differences between communities this indicated the appropriateness of fitting multilevel model.

From the final model, odds of unmet need were 62% [AOR: 1.62, CI (1.25, 2.11)] higher for those females who were heads of the households when compared with their counterparts. Moreover, the current study depicted that odd of unmet need among individual with primary level of education was 1.53 [AOR: 1.53, CI: (1.16, 2.03)] times higher when compared to those with no formal education. The odds of unmet need were 28% [AOR:0.72, CI (0.53, 0.98)] and 42% [AOR: 0.58, CI: (0.40, 0.85) lower among young women who initiated sex at the age of 15–17 years and >17 years as compared to women started sex before the age of 15 years respectively.

The family size was also obtained to have a significant effect on unmet needs for contraceptive. Accordingly, being from the family size ≥5 was found to increase the odds of unmet need by 1.53 [AOR: 1.53, CI: (1.22,1.93)] times when compared with their counterparts. The likelihood of unmet need for contraception among families who were undecided about having more children was 2.86 times higher [AOR: 2.86, CI: (1.58, 5.20)] compared to their counterparts. Additionally, the odds of unmet need among families who were infecund was 1.54 times higher [AOR: 1.54, CI: (1.08, 2.20)] compared to families desiring more children. At the community level, poverty level was significantly associated with unmet contraceptive needs. Women from communities with a high proportion of poverty were 32% less likely [AOR: 0.68, CI: (0.46, 0.99)] to have unmet needs compared to those from communities with a low proportion of poverty (Table 4).

**Table 4:** Determinant associated with unmet need among married women in Ethiopia based on DHS evidence in 2016.

**Variables**	**Model I**	**Model II**	**Model III**	**P-value**
	**AO (95% CI)**	**AO (95% CI)**	**AO (95% CI)**	
**Sex of household head**				
Male	Ref.		1	
Female	1.62(1.25, 2.11)		(1.31,2.25)	0.000
**Wealth quantile**				
Poorest	Ref.		1	
Poorer	0.75(0.53, 1.05)		0.70(0.49,1.01)	0.059
Middle	0.72(0.49, 1.04)		0.73(0.48, 1.10)	0.136
Richer	0.57(0.37, 0.86)		0.61(0.37, 1.00)	0.050
Richest	0.48(0.32, 0.71)		0.50(0.27, 0.94)	0.032
**Women’s educational status**				
No education	Ref.		1	
Primary	1.72(1.31, 2.26)		1.53(1.16,2.03)	0.003
Secondary and above	1.62(1.09, 2.39)		1.35(0.90,2.04)	0.150
**Partners employment**				
Not working	Ref.		Ref.	
Working	0.71(0.49, 1.02)		0.74(0.51,1.08)	0.118
**Age at first sex**				
<15	Ref.		Ref.	
15–17	0.71(0.52, 0.96)		0.72(0.53, 0.98)	0.036
> = 18	0.58(0.40, 0.85)		0.58(0.40, 0.85)	0.006
**Family size**				
<5	Ref.		Ref.	
> = 5	1.54(1.22, 1.95)		1.53(1.22,1.93)	0.000
**Age at first birth**				
Not given birth	Ref.		Ref.	
<18 years	0.94(0.68, 1.29)		0.88(0.64, 1.21)	0.423
> = 18 years	1.06(0.80, 1.42)		1.03(0.77, 1.37)	0.853
**Respondents desire for more children**				
Has desire	Ref.		1	
Undecided	2.83(1.55, 5.17)		2.86(1.58, 5.20)	0.001
Wants no more or infecund	1.70(1.19, 2.42)		1.54(1.08, 2.20)	0.017
**Media exposure**				
Not exposed	Ref.		1	
Exposed	0.84(0.64, 1.11)		0.82(0.61, 1.11)	0.191
***Community level variable***				
**Community Wealth**				
Low		Ref.	Ref.	
High		0.53(0.39,0.73)	0.68(0.46, 0.99)	0.044
**Place of residence**				
Urban		Ref.	Ref.	
Rural		0.95(0.641.40)	0.74(0.43, 1.28)	0.278
**Administrative region**				
Tigray		Ref.	Ref.	
Afar		1.04(0.63,1.72)	0.80(0.47,1.37)	0.412
Amhara		0.75 (0.42, 1.32)	0.74(0.42,1.33)	0.315
Oromia		1.69 (1.04, 2.76)	1.64(0.99,2.70)	0.053
Somali		0.65 (0.37, 1.14)	0.61(0.34,1.09)	0.096
Benishangul		1.39 (0.81, 2.39)	1.33(0.76,2.31)	0.316
SNNPR		1.19 (0.70, 2.03)	1.26(0.73,2.16)	0.411
Gambela		2.04 (1.20,3.45)	1.48(0.85,2.56)	0.166
Harari		1.42 (0.79, 2.55)	1.60(0.88,2.90)	0.122
Addis adaba		0.42 (0.16, 1.11)	0.45(0.17,1.20)	0.109
Dire dawa		0.91 (0.47,1.78)	0.91(0.46,1.80)	0.793
**Community media exposure**				
Low		Ref.	Ref.	
High		1.01 (0.76, 1.34)	1.16(0.84, 1.59)	0.368
**Model parameter**	**Null model**	**Model I**	**Model II**	**Model III**
ICC	0.135	0.107	0.082	0.075
PCV	Reference	0.207	0.393	0.444
Log likelihood	-1154.99	-1106.25	-1127.16	-1090.67
AIC	2313.98	2248.50	2284.32	2243.34
BIC	2325.59	2352.92	2371.34	2423.18

## Discussion

This study was intended to assess the determinants of unmet need for contraceptive method among young women using multi-level analysis. Accordingly, about one-fifth (18.4%) of young married women have unmet need for family planning. Furthermore, 89.3% and 10.7% have unmet need for spacing and limiting respectively. This corresponds to other research findings that indicate a higher prevalence of unmet need for birth spacing compared to unmet need for birth limiting [11, 18, 20]. However, the overall prevalence is lower than pocket studies reported in Ethiopia 25.4% [29], 34.6% [18] and Nepal 38.2% [11]. The observed difference might be attributed to the variation in the study settings, study periods and characteristics of the study populations. Moreover, it could be due to the improvements in the convergence of demand with supply and contraceptive methods utilization in the current study.

In this study, the odd of unmet need for family planning was 1.62 times more likely among young women in female-headed households as compared to those in male-headed households. This is supported by the studies from India [14] and Nigeria [30] which reported that the unmet need for family planning was high among the women who were the head of the households. This might be due to the high need for family planning for a woman who is head of household as she is more responsible and has less time for childbearing.

The odds of unmet need for family planning in this study are 1.53 times higher for women with primary education as compared to those with no formal education. Although it is similar to the study in Nigeria [30], it is inconsistent with other study findings from Ethiopia and Ghana in which unmet need for family planning was less likely for women who attained primary and secondary education as compared to those with no formal education [26, 31–33]. A possible reason for the discrepancy might be due to the difference in study populations. Furthermore, the increased unmet need among the educated may arise from the increased demand for family planning by those groups [34].

This study also revealed that the odds of unmet need for family planning among young married women reduced as age at first sexual intercourse increased by one year. Similarly, the study from Nepal also indicated that the age of the woman was significantly associated with the unmet need for family planning [11]. This might be attributed to the high demand for contraception for younger women to prevent pregnancy-related complications and unsafe abortions among younger women.

The unmet need for family planning increased with increased family size in this study. This finding is in line with the studies in Ethiopia [4, 20, 32, 35], Ghana [31], Nigeria [30], and other low and middle-income countries [36] in which the odds of unmet need for family planning was higher for the woman who had many living children. This may occur when a woman has a satisfied need for additional children and an increased need for family planning which may be left unmet. Additionally, women may have many children because of unmet need for family planning.

Moreover, the unmet need for family planning was found to be decreased with increased desire for more children. This is supported by the study conducted in Eastern Ethiopia which reported that the unmet need for contraception was higher among young married women who desire less than or equal to five children as compared to those who desire greater than five children [18]. Further, each additional child the married young women desired to have decreased the likelihood of the unmet need for contraception by 30% according to the study from Burkina Faso and Mali [23]. The reason behind this may be that as young women desire many children, their need for family planning is reduced and leads to low unmet need for contraceptive methods.

Regarding community poverty level, unmet need among the high poverty level is found to be 32% less likely when compared to women from low poverty level. This is supported by evidence reported from Ghana [7, 31, 37]. However, it is inconsistent with the study conducted in Nepal [11] and Nigeria [30]. This might be due to the fact that women from higher socioeconomic status have more resources and flexibility to access family planning methods and satisfy their reproductive health needs compared to those with lower socioeconomic status.

### Strength and limitations of the study

The study had some inherent limitations worth acknowledging. Given that the unmet need for contraceptives arises from factors like awareness gaps, personal beliefs, family attitudes, fear of side effects, and inconvenience, the subject is more qualitative in character and necessitates more in-depth research than can be accomplished through quantitative analysis. Because this study was based on secondary data, the authors had no control over the study’s design and were unable to select variables based on their demonstrated relationship with unmet family planning needs. Furthermore, unmet need data were self-reported and are prone to reporting bias. The demographic and health surveys were cross-sectional, meaning the result and explanatory variables were measured at the same time, no causation of the link could be established. Nonetheless, the study used nationally representative data and hence the findings are generalizable to all young women in Ethiopia.

## Conclusion

In this study, the magnitude of unmet need for contraceptive methods among young married women was found to be high. The study also found that the factors affecting the family planning need are both the individual and community level factors. Furthermore, being a female household head, age at first sexual intercourse, educational status of the woman, family size, desire for more children, and poverty level are the individual and community level factors influencing the unmet need for a contraceptive method. This indicates that there is a need for emphasizing both the individual and the community to tackle the problem of unmet contraception needs. Therefore, appropriate individual and community level interventions should target young women to address their unmet family planning needs and to avert the contributing factors of unmet family planning needs. All concerned bodies should promote the use of contraceptives among women with unmet family planning needs which is indeed a critical strategy for reducing unsafe abortion, miscarriage, and maternal and infant death.

## Supporting information

S1 File Extracted data for unmeet of FP. (DTA)

## Abbreviations

AIC: Akaike information criteria
AOR: Adjusted Odds Ratio
BIC: Bayesian Information Criteria
COR: Crude Odds Ratio
PCV: proportional change in variance
SPSS: Statistical Package for Social Science

## References

[1] Unmet need for family planning [https://www.data4impactproject.org/prh/family-planning/fp/unmet-need-for-family-planning/]

[2] Unmet need for Family Planning. https://www.who.int/reproductivehealth/topics/family_planning/unmet_need_fp/en/.

[3] Csa I: Central statistical agency (CSA)[Ethiopia] and ICF. *Ethiopia demographic and health survey*, *Addis Ababa*, *Ethiopia and Calverton*, *Maryland*, *USA* 2016, 1.

[4] Ayele W, Tesfaye H, Gebreyes R, Gebreselassie T: Trends and determinants of unmet need for family planning and programme options, Ethiopia. *Further analysis of the* 2000 2005, 2011.

[5] Adolescent health in the South-East Asia Region [https://www.who.int/southeastasia/health-topics/adolescent-health]

[6] Mulenga JN, Bwalya BB, Mulenga MC, Mumba K: Determinants of unmet need for family planning among married women in Zambia. *Journal of Public Health in Africa* 2020, 11(1). doi: 10.4081/jphia.2020.1084 33209230 PMC7649728

[7] Nzokirishaka A, Itua I: Determinants of unmet need for family planning among married women of reproductive age in Burundi: a cross-sectional study. *Contraception and reproductive medicine* 2018, 3:1–13. 29951222 10.1186/s40834-018-0062-0 PMC6011199

[8] Indicators K: Mini demographic and health survey. *EPHI and ICF* 2019.

[9] Sustainable Development Goals. http://www.un.org/sustainabledevelopment/health/. Accessed on 29/9/2021.

[10] Sedgh G, Ashford LS, Hussain R: Unmet need for contraception in developing countries: examining women’s reasons for not using a method. 2016.

[11] Lamichhane K: Unmet need for family planning among currently married young women in Nepal. *Journal of Development and Administrative Studies* 2017, 25(1–2):83–94.

[12] Bishwajit G, Tang S, Yaya S, Feng Z: Unmet need for contraception and its association with unintended pregnancy in Bangladesh. *BMC pregnancy and childbirth* 2017, 17(1):1–9. 28606062 10.1186/s12884-017-1379-4 PMC5469146

[13] Ahinkorah BO, Ameyaw EK, Seidu A-A: Socio-economic and demographic predictors of unmet need for contraception among young women in sub-Saharan Africa: evidence from cross-sectional surveys. Reproductive health 2020, 17:1–11. 33097088 10.1186/s12978-020-01018-2 PMC7585192

[14] Singh LM, Prinja S, Rai P, Siddhanta A, Singh AK, Sharma A, et al: Determinants of modern contraceptive use and unmet need for family planning among the urban poor. *Open Journal of Social Sciences* 2020, 8(5):451–473.

[15] Sinai I, Nyenwa J, Oguntunde O: Programmatic implications of unmet need for contraception among men and young married women in northern Nigeria. *Open Access Journal of Contraception* 2018:81–90. doi: 10.2147/OAJC.S172330 30519126 PMC6236097

[16] Mahuro G, Kimani M: Inequities in unmet need for contraception among married women: Evidence from the PMA2020/Kenya survey. *Cogent Medicine* 2021, 8(1):1943125.

[17] Islam AZ, Mostofa MG, Islam MA: Factors affecting unmet need for contraception among currently married fecund young women in Bangladesh. *The European Journal of Contraception & Reproductive Health Care* 2016, 21(6):443–448. doi: 10.1080/13625187.2016.1234034 27676285

[18] Dingeta T, Oljira L, Worku A, Berhane Y: Unmet need for contraception among young married women in eastern Ethiopia. *Open Access Journal of Contraception* 2019:89–101. doi: 10.2147/OAJC.S227260 31908548 PMC6925555

[19] Tahir Yousufa OMA, Nega Assefac, Tesfaye Gobanad, Pe Lathwal O THE MAGNITUDE AND FACTORS ASSOCIATED WITH UNMET NEED FOR FAMILY PLANNING AMONG MARRIED WOMEN IN JIJIGA CITY ADMINISTRATION, SOMALI REGION, EASTERN ETHIOPIA. *International Journal of Sciences*: *Basic and Applied Research (IJSBAR)* 2019, 43.

[20] Hailemariam A, Haddis F: Factors affecting unmet need for family planning in southern nations, nationalities and peoples region, Ethiopia. *Ethiopian journal of health sciences* 2011, 21(2):77–90. doi: 10.4314/ejhs.v21i2.69048 22434988 PMC3275860

[21] Goodkind D, Lollock L, Choi Y, McDevitt T, West L: The demographic impact and development benefits of meeting demand for family planning with modern contraceptive methods. *Global health action* 2018, 11(1):1423861. doi: 10.1080/16549716.2018.1423861 29415632 PMC5814765

[22] Ameyaw EK, Budu E, Sambah F, Baatiema L, Appiah F, Seidu A-A, et al: Prevalence and determinants of unintended pregnancy in sub-Saharan Africa: A multi-country analysis of demographic and health surveys. *PloS one* 2019, 14(8):e0220970. doi: 10.1371/journal.pone.0220970 31398240 PMC6688809

[23] O’Regan A, Thompson G: Indicators of young women’s modern contraceptive use in Burkina Faso and Mali from Demographic and Health Survey data. *Contraception and reproductive medicine* 2017, 2:1–8. doi: 10.1186/s40834-017-0053-6 29201431 PMC5683538

[24] Girma Garo M, Garoma Abe S, Dugasa Girsha W, Daka DW: Unmet need for family planning and associated factors among currently married women of reproductive age in Bishoftu town, Eastern Ethiopia. *PLoS One* 2021, 16(12):e0260972. doi: 10.1371/journal.pone.0260972 34871318 PMC8648111

[25] Terefe G, Abebe F, Teka B: Unmet Need for Family Planning Service and Associated Factors Among Homeless Women of Reproductive Age Group in Jimma Zone Administrative Towns, Ethiopia. *Open Access J Contracept* 2022, 13:83–93. doi: 10.2147/OAJC.S363258 35702259 PMC9188778

[26] Getaneh T, Negesse A, Dessie G, Desta M, Moltot T: Predictors of unmet need for family planning in Ethiopia 2019: a systematic review and meta analysis. *Archives of Public Health* 2020, 78:1–11. 33088503 10.1186/s13690-020-00483-2 PMC7566059

[27] Teshale AB: Factors associated with unmet need for family planning in sub-Saharan Africa: A multilevel multinomial logistic regression analysis. *PloS one* 2022, 17(2):e0263885. doi: 10.1371/journal.pone.0263885 35143584 PMC8830726

[28] Tricco AC, Lillie E, Zarin W, O’Brien KK, Colquhoun H, Levac D, et al: PRISMA extension for scoping reviews (PRISMA-ScR): checklist and explanation. *Annals of internal medicine* 2018, 169(7):467–473. doi: 10.7326/M18-0850 30178033

[29] Mekonnen W, Worku A: Determinants of low family planning use and high unmet need in Butajira District, South Central Ethiopia. *Reproductive health* 2011, 8:1–8. 22151888 10.1186/1742-4755-8-37 PMC3248357

[30] Oginni AB, Ahonsi BA, Adebajo S: Trend and determinants of unmet need for family planning services among currently married women and sexually active unmarried women aged 15–49 in Nigeria (2003–2013). *African Population Studies* 2015, 29(1):1483–1499.

[31] Nyarko SH, Sparks CS, Bitew F: Spatio-temporal variations in unmet need for family planning in Ghana: 2003–2014. *Genus* 2019, 75(1):1–13.

[32] Alem AZ, Agegnehu CD: Magnitude and associated factors of unmet need for family planning among rural women in Ethiopia: a multilevel cross-sectional analysis. *BMJ open* 2021, 11(4):e044060. doi: 10.1136/bmjopen-2020-044060 33837100 PMC8043003

[33] Tegegne KT, Kifle Y, Belayneh T, Tegegne ET, Tessema MK: Unmet Need for Family Planning Among Women of Reproductive Age Living in Shebedeno. 2021.

[34] Hosseini H, Torabi F, Bagi B: Demand for long-acting and permanent contraceptive methods among Kurdish women in Mahabad, Iran. *Journal of biosocial science* 2014, 46(6):772–785. doi: 10.1017/S0021932013000710 24406051

[35] Yalew M, Adane B, Kefale B, Damtie Y: Individual and community-level factors associated with unmet need for contraception among reproductive-age women in Ethiopia; a multi-level analysis of 2016 Ethiopia Demographic and Health Survey. *BMC Public Health* 2020, 20(1):1–9. 32306933 10.1186/s12889-020-08653-1 PMC7168822

[36] Wulifan JK, Brenner S, Jahn A, De Allegri M: A scoping review on determinants of unmet need for family planning among women of reproductive age in low and middle income countries. *BMC women’s health* 2015, 16:1–15. 10.1186/s12905-015-0281-3 PMC4714507 26772591

[37] Out-look 25th Anniversary Issue. 2008 [https://www.unfpa.org/sites/default/files/resource-pdf/EOL_nov08.pdf]

